# Enrichment and analysis of secretory lysosomes from lymphocyte populations

**DOI:** 10.1186/1471-2172-10-41

**Published:** 2009-07-29

**Authors:** Hendrik Schmidt, Christoph Gelhaus, Ralph Lucius, Melanie Nebendahl, Matthias Leippe, Ottmar Janssen

**Affiliations:** 1 Molecular Immunology, Institute of Immunology, University Hospital Schleswig-Holstein Campus Kiel, Kiel, Germany; 2 Department of Zoophysiology, Zoological Institute, Christian-Albrechts-University, Kiel, Germany; 3 Institute of Anatomy, Christian-Albrechts-University, Kiel, Germany

## Abstract

**Background:**

In specialized cells, such as mast cells, macrophages, T lymphocytes and Natural Killer cells in the immune system and for instance melanocytes in the skin, secretory lysosomes (SL) have evolved as bifunctional organelles that combine degradative and secretory properties. Mutations in lysosomal storage, transport or sorting molecules are associated with severe immunodeficiencies, autoimmunity and (partial) albinism. In order to analyze the function and content of secretory lysosomes in different cell populations, an efficient enrichment of these organelles is mandatory.

**Results:**

Based on a combination of differential and density gradient centrifugation steps, we provide a protocol to enrich intact SL from expanded hematopoietic cells, here T lymphocytes and Natural Killer cells. Individual fractions were initially characterized by Western blotting using antibodies against an array of marker proteins for intracellular compartments. As indicated by the presence of LAMP-3 (CD63) and FasL (CD178), we obtained a selective enrichment of SL in one of the resulting organelle fractions. The robustness and reproducibility of the applied separation protocol was examined by a high-resolution proteome analysis of individual SL preparations of different donors by 2D difference gel electrophoresis (2D-DIGE).

**Conclusion:**

The provided protocol is readily applicable to enrich and isolate intact secretory vesicles from individual cell populations. It can be used to compare SL of normal and transformed cell lines or primary cell populations from healthy donors and patients with lysosomal storage or transport diseases, or from corresponding mutant mice. A subsequent proteome analysis allows the characterization of molecules involved in lysosomal maturation and cytotoxic effector function at high-resolution.

## Background

Cytotoxic T lymphocytes (CTL) and Natural Killer (NK) cells deliver their cytolytic effector molecules via so-called secretory lysosomes (SL). These organelles contain a number of cytotoxic proteins and undergo regulated secretion in response to external stimuli, for example to eliminate virus-infected and tumorogenic cells by the induction of apoptosis. In most eukaryotic cells, lysosomes are acidic organelles equipped with hydrolases to degrade and recycle endogenous or exogenous biomolecules [1]. Interestingly, in various hematopoietic cell lineages associated with secretory effector functions (CTL, NK, mast cells and macrophages), a subset of lysosomes gained a second function as a storage and transport vehicle for effector molecules [2,3]. Similarly, outside the immune system, melanocytes utilize a comparable organelle to regulate pigmentation [4].

In mast cells, histamine and serotonin are the key factors associated with SL, whereas in macrophages, for example MHC molecules and cytokines are stored and transported via this compartment [2]. As mentioned, SL of mature T and NK cells are characterized by the presence of cytotoxic effector molecules including perforin [5], granzymes [5], granulysin [6], and the TNF family protein FasL (CD178) [7,8]. Interestingly, the 'packaging' of individual effector molecules is quite different. Granzymes and perforins appear to be stored at least in part in association with carrier proteins (e.g. serglycins) in the vesicular lumen to form dense core particles [9]. In contrast, the FasL is a transmembrane protein that in fact might be 'directly' involved in the transport of SL during organelle maturation and effector responses. During maturation, FasL is transported to SL by SH3 domain proteins interacting with its proline-rich C-terminus. Subsequently, FasL might participate in connecting SL to the actin-cytoskeleton via its interaction with Nck and associated actin-regulatory proteins [10-12].

Genetic diseases based on defects of the secretory machinery such as the Chédiak-Higashi Syndrome (CHS), the Hermansky-Pudlak Syndrome (HPS) or the Griscelli Syndrome (GS) are characterized by a combination of immunodeficiency and albinism, reflecting the close relationship of the SL-related secretory pathway in hematopoietic cells and melanocytes as non-hematopoietic cells [4]. Although the genetic defects have been mostly deciphered, their influence on the overall protein composition of secretory lysosomes is not completely understood. Of note, a number of mouse strains are available that reflect the pathophysiology in patients. As an example, LYST-deficient mice are the murine counterpart of CHS and thus suffer from immunodeficiency and also carry a respective fur phenotype ('beige mouse'). Cells of such mice harbor giant lysosomes, indicating that the lysosomal maturation and storage might still be operational, while exocytosis and recycling are severely impaired [13].

For the detailed analysis of the proteomic profile of SL, it is important to efficiently enrich or purify this compartment from a limited number of cells. In 1978, a first density gradient centrifugation protocol was developed to purify organelles from rat hepatocytes using a non-ionic X-ray contrast compound termed Metrizamide [14]. Compared to traditional sucrose gradients, the advantage of Metrizamide was a much lower osmolarity that enabled adequate separation of lysosomes and mitochondria without artificially altering their density as for instance observed with Triton WR1339 [15] or dextran [16]. In the meantime, several non-ionic iodinated density gradient media have been developed, which are often derivatives of metrizoic acid, for example Nycodenz and its dimer Iodixanol (commercially available as OptiPrep™, a 60% solution of Iodixanol-5,5'- [(2-hydroxy-1,3-propanediyl)-bis(acetylamino)]bis [N, N'-bis(2,3-dihydroxypropyl-2,4,6-triiodo-1,3-benzenecarboxamide). The major advantage of Iodixanol in this context is its ability to form iso-osmotic solutions at high densities (up to 1.32 g/ml) [17]. In 1994, Graham et al. first described the usage of Iodixanol as density gradient media and provided a first set of subcellular isolation protocols [18,19].

Another standard gradient medium that also offers lower osmolarity and viscosity is Percoll™. It consists of colloidal silica coated with polyvinylpyrrolidone (PVP). In the last three decades, Percoll™ gradients were not only used to separate lymphocyte subpopulations but also to isolate cytotoxic granules of NK [20,21] and T cell lines [21,22]. Recently, several Percoll-based isolation protocols were compared with regard to the functionality of effector molecules in the obtained fractions in cytotoxicity assays [23]. A major disadvantage of Percoll™ as a separation medium is that silica often has to be removed from the obtained fractions because it might interfere with downstream applications. Unfortunately, however, such washing steps are mostly accompanied by the loss of organelle integrity.

Here, we describe a rapid and reliable Iodixanol-based subcellular fractionation protocol that allows the substantial enrichment of intact secretory vesicles for example from *in vitro *expanded lymphocyte populations. Importantly, the obtained fractions are compatible with standard analyses such as Western blotting and even with CyDye labeling for subsequent high-resolution two-dimensional difference gel electrophoresis [24].

## Results

### Subcellular fractionation

**Figure 1:**
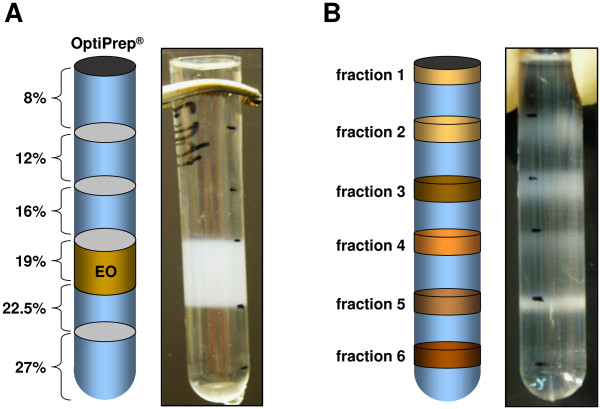
**Purification of enriched secretory lysosomes**. A. Enriched organelle fractions (EO) were adjusted to 19% (v/v) OptiPrep^® ^and loaded in the middle of the discontinuous density gradient. B. After 5 h of ultracentrifugation, six individual fractions became apparent at the individual interphases that were collected, pelleted and used for subsequent analyses.

**Figure 2:**
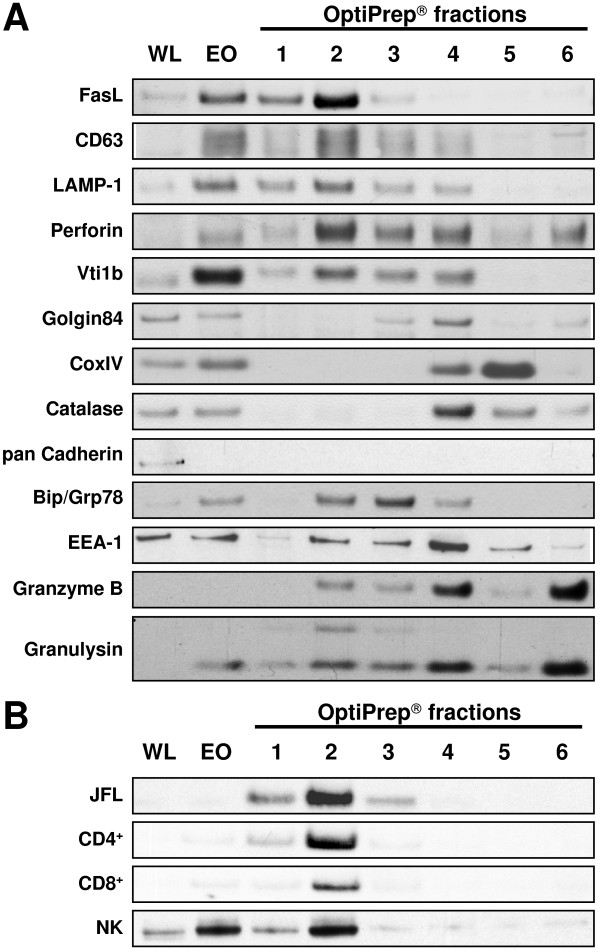
**Representation of marker proteins in individual OptiPrep^® ^fractions**. A. The individual lanes represent whole cell lysate (WL), enriched organelles (EO) and six individual fractions collected after density gradient centrifugation of PHA blasts expanded for 14 days. The blots were analyzed for the presence of known organelle marker proteins as indicated. One representative Western blot of four performed experiments with different T cell blasts is shown. B. An enrichment of FasL (as a marker protein for secretory lysosomes) is detected in fraction 2 of different lymphocyte subpopulations including CD4^+ ^and CD8^+ ^T cells, in vitro expanded human NK cells and the stably FasL-transfected Jurkat variant JFL.

For the purification of lysosomal fractions, we applied a low osmotic medium for density gradient centrifugation. Following primary organelle enrichment by differential centrifugation, the subcellular compartments were separated on a discontinuous Iodixanol gradient (Figure 1A). Individual fractions were collected from the top of the gradient and the protein content of the six obtained fractions (Figure 1B) was quantified using a Bradford protein assay. Equal amounts of protein were then separated by SDS-PAGE and analyzed by Western blotting for the presence of characteristic organelle marker proteins (Figure 2). In order to define the major lysosomal fraction, Western blots were stained for CD63 (LAMP-3 or LIMP) and CD107a (LAMP-1) as classical lysosome-associated membrane proteins and for CD178 (FasL) and perforin as characteristic marker proteins for secretory lysosomes of T and NK cells [3,25]. In addition, we stained for Vti1b, a membrane-bound SNARE protein that is thought to function in the regulation of post-Golgi vesicle trafficking and secretory pathways [26]. As detailed in Figure 2A, all putative marker proteins for secretory lysosomes and also Vti1b were enriched in fraction 2 of the final Iodixanol gradient. At the same time, this putative secretory lysosomal fraction 2 did not contain Golgin84, a marker for Golgi cisternae [27], or CoxIV as a marker for mitochondria [28], or catalase as an indicator for peroxisomes [29]. Furthermore, all Iodixanol fractions were not stained with an anti-pan-cadherin antibody indicating that they are devoid of contaminating plasmamembrane fragments that were readily removed during the first differential centrifugation step. It should be mentioned, however, that organelle separations by density gradients should not be regarded as purifications but rather as enrichment procedures. Following our protocol, we thus found Bip/Grp78, a marker for the endoplasmic reticulum [30], and EEA-1, a marker for early endosomes [31] as 'contaminants' in the putative lysosomal fraction 2. Interestingly, granzyme B and granulysin, two cytotoxic effector molecules that are known to be present in secretory vesicles [5,6] were also present in other fractions. In case of granulysin, the long (premature) form of 15 kDa was selectively enriched in fraction 2 whereas the mature form (9 kDa) was detected in all fractions with the highest abundance in fractions 4 and 6. Similarly, granzyme B was detected in fractions 2–6, again with the strongest signals after Western blotting in fractions 4 and 6. Most importantly, however, if putative SL were enriched from different FasL-containing cell populations, including stable Jurkat transfectants (JFL), *in vitro *expanded CD4^+ ^and CD8^+ ^T cells, or NK cells, we consistently noted a substantial enrichment of this marker protein in the respective fraction 2 (Figure 2B).

### Electron micrographs of isolated vesicles

**Figure 3:**
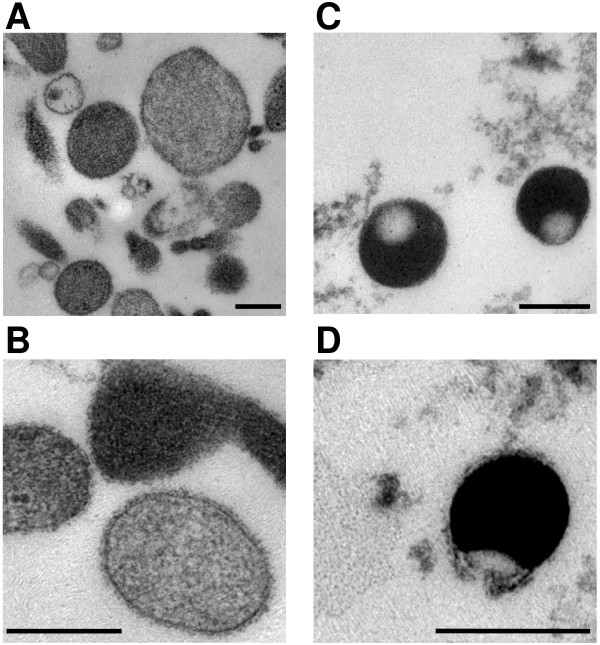
**Electron micrographs of isolated secretory granules**. Enriched granules of PHA blasts corresponding to fraction 2 (A, B) or fraction 6 (C, D) were fixed and embedded in araldite. Overview pictures are given in A and C, higher magnifications in B and D. (Bar, 250 nm)

To verify whether the obtained fractions contain intact vesicles, isolated granules of 13 days old PHA blasts corresponding to fractions 2 and 6 were fixed and processed for electron microscopical inspection as detailed in the Material and Methods section. As shown in Figure 3, round membrane-enclosed organelles with a characteristic electron density and a diameter between 250 and 700 nm formed a predominant organelle population in fraction 2 (Figure 3A, B). In contrast, the predominant structures in fraction 6 were slightly smaller, membrane-enclosed and somehow polar vesicles that were mainly characterized by a much higher electron density (Figure 3C, D).

### 2D-DIGE of enriched secretory granules

**Figure 4:**
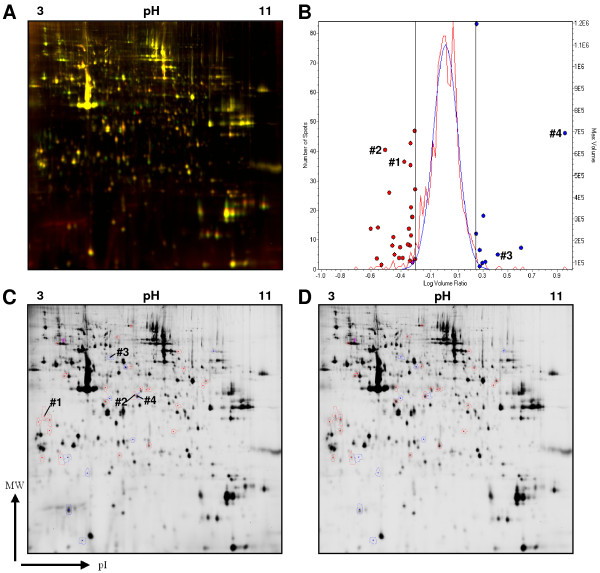
**2D-DIGE analysis of enriched SL of PHA blasts from two different donors**. A. Fifty μg of fraction 2 protein were labeled with Cy3 (donor 1, green channel) or Cy5 (donor 2, red channel), respectively and spiked with unlabelled protein. Yellow indicates comparable abundance of proteins in both samples. One representative experiment of two performed with unseparated PHA blasts is shown – more experiments were performed with lymphocytes subpopulations. B. Statistical representation of the overall protein distribution in individual samples. The red line indicates the number of spots with a specific abundance ratio (log scale). The blue line represents the calculated Gaussian distribution. Red dots represent decreased and blue increased protein content in individual spots detected in both samples (considered from Cy3 → Cy5). The threshold was set to 1.7. C, D. Individual images corresponding to the individual samples of donor 1 (C) and donor 2 (D), respectively. Differential spots are marked either red or blue reflecting the color-coding in B. The numbers displayed in B and C correspond to the numbers in Figure 5 and table 1.

In order to analyze the robustness of our purification protocol, PHA-stimulated lymphocytes of two healthy donors were expanded *in vitro *for 16 days. After differential and density gradient centrifugation, the obtained organelles of fraction 2 were pelleted and lyzed in 2D lysis buffer. For the subsequent 2D-DIGE analysis, 50 μg of enriched SL (= fraction 2) of donor 1 were labeled with Cy5 and 50 μg of SL of donor 2 with Cy3. The samples were combined, spiked with 50 μg each of unlabeled fraction 2 protein and subjected to isoelectric focusing on an IPG strip (pH 3–11 NL) in the first dimension and a conventional SDS-PAGE (12.5% gel) for the second dimension. A characteristic protein distribution along with the initial analysis using the DeCyder^® ^software package is shown in Figure 4. As indicated by the yellow color in the overlay (Figure 4A), the two fractions were extremely similar although the original cells were derived from two different donors, expanded for more than two weeks and both samples were isolated on separate gradients. The high reproducibility of the lysosomal enrichment using individual biological samples is also reflected in the graphical depiction of the spot distribution (Figure 4B). Using standard settings, of the 972 included proteins, 34 were marked different with a threshold set to 1.7 fold and only 7 with a threshold set to 3 fold. Of note, a threshold of 1.7 is equivalent to a two-fold square deviation of the spot volumes and results in a variance of approximately 3.5% of the calculated protein content.

### Peptide mass fingerprinting of selected spots

**Figure 5:**
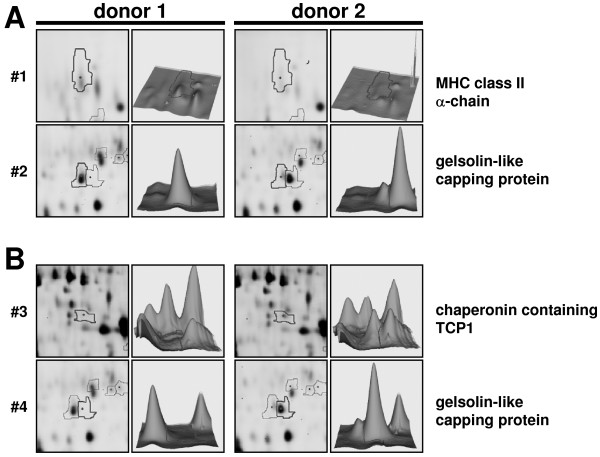
**Selected differential spots in SL preparations of two different donors**. 3D images of differentially expressed proteins in the lysosomal preparations of donor 1 and donor 2. Two proteins (#1: MHC class II alpha chain and #2: capping protein (actin filament), gelsolin-like) are reduced in donor 2 (A) and two proteins (#3: chaperonin containing TCP1, subunit 8 and #4: capping protein (actin filament), gelsolin-like) are reduced in donor 1 (B). The bold grey line indicates the same location in both images. The intensity of the spot is indicated by the 3D views. Spots #2 and #4 presumably represent isoforms of the gelsolin-like capping protein.

In order to identify selected differentially represented spots by peptide mass fingerprinting (PMF), individual spots were automatically picked using an Ettan Spotpicker. Tryptic digestion was performed as described before [32] and eluted peptides were dried, resuspended in MALDI matrix and spotted on MALDI targets. The subsequent PMF analyses revealed differences for example in MHC class II molecules (#1; α-chain -2.13 fold), gelsolin-like capping protein isoforms (#2; -3.03 and #4; 8.98 fold) and in chaperonin containing TCP1, subunit 8 (#3; 2.63 fold) between the two samples (Table 1 and Figure 5).

**Table 1:** Selected proteins identified by peptide mass fingerprinting.

**spot #**	**protein name**	**Vol. ratio**	**NCBI Acc.No**.	**MW**	**pI**	**identified peptides**	**MASCOT score**
1	MHC class II alpha chain	-2.13	gi|3212400	20548	4.9	3	113
2	capping protein (actin filament), gelsolin-like	-3.03	gi|63252913	38760	5.8	9	334
3	chaperonin containing TCP1, subunit 8	2.63	gi|48762932	60153	5.4	5	183
4	capping protein (actin filament), gelsolin-like	8.98	gi|55597035	38779	5.9	9	368

### Similarity of individual SL preparations

**Figure 6:**
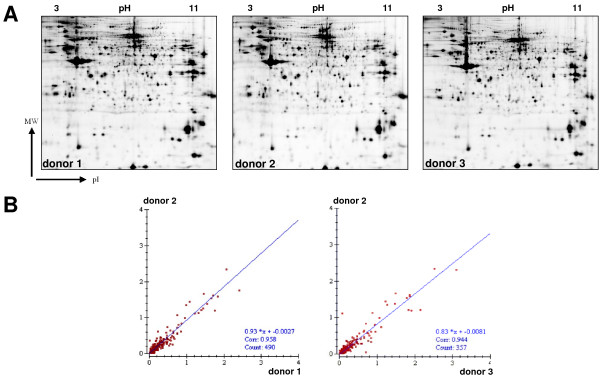
**Similarity of protein content in SL of different CD4^+ ^cell lines**. A. Isolated SL (fraction 2) of CD4^+ ^cells from 3 different donors were separated on individual gels and stained with Flamingo Pink. B. Statistical analysis of spot intensities between the 3 donors using Image Master software revealed a highly similar protein pattern as indicated by the regression line and the correlation coefficient.

We also wanted to know whether enriched secretory lysosomes of individual T cell subpopulation show a comparable protein pattern. To this end, we isolated CD4^+ ^T cells of three different donors by magnetic-activated cell sorting. Individual fraction 2 samples were compared by 2D electrophoresis on separate gels stained with Flamingo Pink. Scanning, data acquisition and individual intra-gel comparisons using the Image Master 6.0 software package again revealed a highly similar protein pattern in all samples (Figure 6). The average correlation coefficient in scatter plot images was 0.95 (Figure 6B). Of note, this coefficient can vary between -1 and 1, with an absolute value of 1 indicating the perfect fit. As a conclusion, the proteomic profiles of enriched fraction 2 SL of separately expanded CD4^+ ^T cell populations of three different donors are indeed very similar with only a few proteins being differentially expressed.

## Discussion

In the present study we describe a reliable protocol for the enrichment of intact secretory vesicles for the subsequent detailed analysis of this subcellular compartment. Combinations of differential and density gradient centrifugation procedures are widely used to isolate organelle fractions. Iodinated, low osmotic media are advantageous in this context not only because of their low osmolarity but also because they lack silica, which might interfere with organelle integrity and subsequent analyses. Since the density of lysosomes might vary depending on the cell or tissue type, an optimization of gradient sizes and concentrations, as well as centrifugation setting is required to obtain reproducible results. We optimized this protocol for the rapid and reproducible enrichment of secretory lysosomes of lymphocyte subpopulations. Using this methodology, we already defined the SL proteome of NK cell lines and primary NK cells [32]. In the present study, we tested the reproducibility and reliability of the procedure by comparing purified SL fractions of polyclonal activated T cell populations (PHA blasts) and subpopulations (CD4^+ ^T cells) of different donors by subsequent Western blotting and high-resolution 2D difference gel electrophoresis. We show that the SL fractions of T cells from healthy donors are very similar with regard to the overall protein content, with only minor differences in MHC molecules or associated transporter proteins or chaperons. Thus, this methodology is apparently ideal as basis for the detailed investigation of consequences of genetic alterations associated with lysosomal transport and storage diseases.

One initial key step of organelle preparation is the disruption of the cells. We applied a mild dounce homogenization and empirically determined the number of strokes for optimal cell opening with minimal damage to organelles and nuclei by trypan blue staining. The first centrifugation step efficiently removed intact cells, nuclei (data not shown) and associated plasma membrane fragments (Figure 2). Since mitochondria are more sensitive to hydrostatic pressure [33], bottom loading of density gradients caused an increase in their density relative to that of lysosomes. As reported before, we also noted that in iodinated density gradient media such as Iodixanol, lysosomes tend to better separate from mitochondria or peroxisomes than in silica-based percoll media [19].

The interphases containing individually enriched organelles can be removed from the top of the gradient or by perforation of the centrifugation tube with a suitable needle. We routinely collected the samples from the top of the tubes. In our study, the individual fractions were analyzed by Western blotting for the presence of marker proteins for secretory lysosomes (CD63 (LAMP-3), CD107a (LAMP-1) and CD178 (FasL)), mitochondria (Cox IV), peroxisomes (catalase), ER (Bip/Grp78), Golgi (Golgin84) and early endosomes (EEA-1). Although contaminations of lysosomal fractions with mitochondria often hamper analyses of lysosomes obtained by density gradient centrifugation, the use of a discontinuous Iodixanol^® ^separation gradient resulted in a clear separation of Cox IV containing mitochondria (fraction 5) and secretory lysosomes characterized by the presence of LAMP-1, CD63 and FasL (fraction 2). Besides the enrichment of FasL and lysosomal membrane proteins, which is indicative for the enrichment of SL in fraction 2, we observed a rather high abundance of other cytotoxic effector molecules (including granzyme B, perforin and granulysin) in fractions 4 and 6 (Figure 2). The presence of effector molecules in separate intact electron dense fraction 6 granules (Figure 3) was unexpected. Employing the separation method described here, we are currently investigating and comparing the composition of the two vesicular structures in different T and NK cell populations in more detail. We think that the distinct SL/granules actually form two separate compartments that might arise from an initial multivesicular complex during vesicular maturation. Fractions 2 and 6 not only differ substantially in the content of effector molecules but also in proteins that regulate cytoskeletal association (Schmidt et al., unpublished). Of note, since the described purification protocol does not imply nucleases and results in intact granules as shown by electron microscopy (Figure 3), enriched SL (and also fraction 6 granules) should be suitable to assay their capacity to directly induce target cell apoptosis by standard DNA fragmentation or chromium release assays as described before by Trapani and colleagues [23].

Importantly, the relatively high number of cells (we routinely started with 400 × 10^6^) needed as an input for the enrichment of secretory vesicles is easily achievable if one wants to analyze leukemic cell lines or murine or human T cell or NK cell populations. To demonstrate the reproducibility and reliability of this technique, we isolated enriched secretory lysosomes of PHA stimulated lymphocytes of two different donors and compared these fractions by 2D-difference gel electrophoresis. This direct comparison at high-resolution revealed an almost identical spot pattern (96.5% at a threshold of 1.7), indicating that the presence and also the abundance of the approximately 1000 individual proteins in these enriched fractions are highly conserved in biological replicates. To stress this point: if one compares the different fractions from one donor, the protein content is completely different (21.4% comparable proteins in fractions 2 and 5 at a threshold of 1.7).

Since we were interested in the few differences between the two samples, we picked four selected spots and identified the proteins by mass spectrometry. Although only 200 μg of total protein were separated on the respective gels, even low abundant spots (#1, #3) were identified. It is important to mention that spot picking was performed from Flamingo Pink stained gels since we and others noted that CyDye minimal labeling results in significant alterations of protein masses, especially of low molecular weight proteins [34]. Since only 3% of a given protein is labeled by the employed minimal labeling procedure, 97% of the protein might be actually located below the initially detected protein spot. Nonetheless, the subsequent PMF analyses revealed differences in MHC class II molecules, gelsolin-like capping protein isoforms and in chaperonin containing TCP1, subunit 8 between the two samples (Figure 5). Without going into further details, these or related proteins have been associated with secretory vesicles in other studies before [35]. In a recent study, we provided a detailed analysis of the proteome of enriched SL from different NK cell lines and primary NK cells [32]. We identified roughly 400 protein spots representing 237 individual proteins. As a proof of principle, although in NK cells the overall protein content of isolated SL was also very similar, we described a heterogeneous profile of functionally relevant proteins in leukemic and primary NK cells.

The reliability of the presented protocol is further stressed by the comparison of SL from CD4^+ ^T cells isolated from three different donors. Of note, most CD4^+ ^T cells also gain cytotoxic potential associated with SL when expanded *in vitro *[11]. As expected from the initial 2D-DIGE experiments on PHA blasts, also standard 2D gel electrophoresis followed by Flamingo Pink staining and indirect comparison of individual gels using Image Master software revealed a very high degree of similarity between the individual fraction 2 preparations (Figure 6). This notion is highly relevant since future proteome analyses need to be standardized and suitable to compare appropriate numbers of samples for statistical analyses [36].

## Conclusion

We describe a reliable, reproducible and easy to perform method for the enrichment of secretory lysosomes. We demonstrate that enriched SL fractions of individual donors or lymphocyte subpopulations are almost identical in their overall protein content. We thus provide a standard protocol for the future comparison of this important compartment in different T and NK cell subpopulations, in untransformed and transformed cells, and in T and NK cell populations of patients or mice suffering from genetically caused lysosomal storage diseases. Furthermore, the protocol might be easily adapted to investigate lysosomes of other cellular origin, including macrophages, mast cells or melanocytes.

## Methods

### Cells

Human peripheral blood mononuclear cells (PBMC) were isolated from buffy coat preparations by Ficoll (1.077 g/ml) density gradient centrifugation. CD4^+^, CD8^+ ^or NK cells were further purified by magnetic-activated cell sorting from PBMC (MACS Cell Isolation Kits, Miltenyi, Bergisch Gladbach, Germany) and adjusted to 10^6 ^cells/ml in RPMI 1640 supplemented with 10% FCS for CD4^+ ^and CD8^+ ^or with 10% heat-inactivated pooled AB human serum for NK cells. All isolated cells were expanded in the presence of a feeder cocktail of irradiated EBV-transformed B cells and PBMCs. For the generation of PHA blasts, PBMC were adjusted to 10^6 ^cells/ml in RPMI 1640 supplemented with 5% heat-inactivated FCS and 0.5 μg/ml phytohemagglutinin A (PHA) (Remel, Lenexa, KS, USA). After 4 days of stimulation, dead cells were removed by Ficoll gradient centrifugation. All cell cultures were maintained at a cell density of 1.5–2.0 × 10^6 ^cells/ml for up to 3 weeks in the presence of 1000 U/ml human recombinant interleukin 2 (rIL-2, Chiron GmbH, Marburg, Germany) for NK cells and 100 U/ml for CD4^+ ^and CD8^+ ^T cells, respectively. All culture media contained penicillin (50 U/ml) and streptomycin (50 μg/ml). Cell culture was performed at 37°C in a humidified atmosphere with 5% CO_2_.

### Subcellular fractionation

For subcellular fractionation and enrichment of secretory lysosomes from PHA-blasts, 4 × 10^8 ^cells were used. The cells were centrifuged, washed once with ice-cold PBS and resuspended in 2.5 ml extraction buffer including a protease inhibitor cocktail (Sigma, St Louis, MO, USA). Gradient media and buffers were purchased as a kit from Sigma or made up according to Graham [19]. The cells were disrupted in a dounce glass homogenizer with a small clearance pestle using 25 strokes. For the initial enrichment of organelles, the homogenates were separated by centrifugation at 1,000 × g for 10 min to pellet nuclei and remaining intact cells. The postnuclear supernatant was sedimented at 20,000 × g for 20 min. The resulting pellet was adjusted to 19% (v/v) Optiprep^® ^(Sigma), loaded in the middle of a non-ionic, low osmotic discontinuous density gradient with 27%, 22.5%, 19%, 16%, 12%, 8% Optiprep^®^, and subjected to an ultracentrifugation at 150,000 × g for 5 h. The osmolarity was adjusted to 290 mOsm with 2.3 M sucrose. The subcellular fractions were collected from the top of the tube, washed and concentrated with HB-Buffer (250 mM sucrose, 10 mM Hepes pH 7.3; and 0.3 mM EDTA) at 150,000 × g for 20 min. All ultracentrifugation steps were carried out at 4°C in Ultra-Clear centrifugation tubes in a swing-out rotor (SW60Ti, Beckman Coulter, Krefeld, Germany). The protein content of the individual fractions was determined using a Coomassie-based protein assay (Pierce, Rockford, IL, USA). Starting with 400 × 10^6 ^cells, homogenized in 2.70 ml of homogenization buffer, the enriched organelle fraction contained about 1.35 mg of protein (table 2). After density gradient centrifugation, the mean protein concentration in individual fractions was 0.324 mg/ml for fraction 1, and 0.398 mg/ml, 0.889 mg/ml, 0.490 mg/ml, 0.905 mg/ml and 0.379 mg/ml for fractions 2 to 6. Corresponding to the volume of individual fractions (200 μl for fractions 1, 5 and 6, 300 μl for fractions 3 and 4, and 400 μl for fraction 2) the recovery was calculated as detailed in table 2.

**Table 2:** Total protein and protein concentrations in individual fractions obtained after differential and density centrifugation.

**fraction**	**Exp. 1**	**Exp. 2**	**Exp. 3**	**Exp. 4**	**mean**	**SD**	**Volume**	**protein**
	[mg/ml]	[mg/ml]	[mg/ml]	[mg/ml]	[mg/ml]		[μl]	[μg]
**homogenate**	3.891	3.222	3.532	3.684	**3.582**	0.282	**2700**	**9671.4**
**enriched organelles (input)**	0.745	0.651	0.729	0.865	**0.747**	0.088	**1800**	**1345.3**
								
**fraction 1**	0.349	0.303	0.347	0.296	**0.324**	0.028	**200**	**64.7**
**fraction 2**	0.430	0.468	0.306	0.389	**0.398**	0.069	**400**	**159.3**
**fraction 3**	0.764	0.909	1.004	0.879	**0.889**	0.099	**300**	**266.7**
**fraction 4**	0.526	0.548	0.480	0.405	**0.490**	0.063	**300**	**147.0**
**fraction 5**	0.925	0.882	0.959	0.852	**0.905**	0.047	**200**	**180.9**
**fraction 6**	0.384	0.431	0.352	0.349	**0.379**	0.038	**200**	**75.8**

**recovery**								**894.4**

### Transmission electron microscopy

Enriched granules of fractions 2 and 6 were fixed with 2.5% glutaraldehyde in PBS at 4°C overnight, washed in PBS, postfixed in 2% OsO_4_, dehydrated in ethanol, and embedded in araldite (Sigma). Thin sections were mounted on formvar-coated grids and double-stained with a saturated solution of uranyl acetate in 70% methanol and with lead citrate. The grids were examined with a Zeiss EM 900 transmission electron microscope.

### SDS-PAGE and Western blotting

For Western blot analysis, 4 μg of protein per fraction were separated by SDS-PAGE on 4–12% gradient Bis-Tris gels (Invitrogen, Karlsruhe, Germany). After blotting to 0.2 μm nitrocellulose membranes (Biometra, Goettingen, Germany), protein loading and efficacy of transfer was checked by reversible staining with Ponceau S (not shown). Membranes were routinely blocked with 5% non-fat dry milk powder or bovine serum albumin in TBST (TBS + 0.05% Tween), respectively. The fractions were analyzed with anti-FasL monoclonal antibody (mAb) clone G-247.4 (1/1000, BD Bioscience, San Diego, CA, USA), anti-LAMP-1 mAb clone 25 (1/500, BD Biosciences), anti-CD63 mAb clone MEM-259 (1/1000, Acris, Hiddenhausen, Germany), anti-Perforin mAb clone B-D48 (1/500, Abcam, Cambridge, UK), anti-Vti1b mAb clone 7 (1/1000, BD Biosciences), anti-Golgin84 mAb clone 26 (1/250, BD Biosciences), anti-CoxIV mAb clone 10G8D12C12 (1/1000, MitoScience, Eugene, OR, USA), anti-granzyme B mAb clone 2C5/F5 (1/2000, BD Biosciences), anti-EEA-1 mAb clone 14 (1/2500, BD Biosciences), anti-Bip/Grp78 mAb clone 40 (1/250, BD Biosciences), anti-pan-Cadherin mAb clone 22744 (1/1000, Abcam), and horseradish peroxidase (HRP)-conjugated goat anti-mouse secondary antibody (1/7500, GE Healthcare, Uppsala, Sweden). For reprobing, membranes were stripped in stripping solution (100 mM 2-mercaptoethanol, 2% SDS, 60 mM Tris) for 30 min at 56°C. The polyclonal antibodies anti-catalase (1/5000, Abcam) and anti-granulysin (1/1000, RnD Systems, Minneapolis, MN, USA) were detected with HRP-conjugated goat anti-rabbit antibodies and donkey anti-goat, respectively. ECL (enhanced chemiluminescence) reagents (GE Healthcare) were used for chemiluminescent detection using Hyper Film (GE Healthcare).

### 2D-DIGE

Pelleted fractions were lyzed on ice for at least 30 min with 30 μl lysis buffer (pH 8.5) containing 7 M urea, 2 M thiourea, 30 mM Tris and 4% CHAPS [37]. The supernatant was removed after centrifugation for 20 min at 20000 × g at 4°C. 50 μg of protein per sample were labeled on ice with 400 pmol of Cy3 or Cy5 (CyDyes, GE Healthcare) for 30 min in the dark. After quenching the labeling reaction with 1 μl lysine (10 mM) for 10 min on ice, the differentially labeled samples were pooled with 50 μg of unlabeled protein of each sample and mixed with a double volume of rehydration buffer (7 M urea, 2 M thiourea, 4% CHAPS, 2% (v/v) IPG buffer pH 3–11 and 2% (w/v) DTT). The mixed samples were applied by cup-loading onto 24 cm IPG non-linear pH 3–11 gel strips, which had been rehydrated at 20°C for at least 10 h in 450 μl DeStreak solution (GE Healthcare) with 0.5% (v/v) IPG buffer pH 3–11 NL. Isoelectric focusing (IEF) was carried out for a total of 50–55 kVh at 20°C in five steps with a gradual increase of voltage (150 V for 3 h, 300 V for 4 h, gradient from 300 V to 1000 V for 6 h, gradient from 1000 V to 10000 V for 3.5 h and 10000 V ~3 h) using the IPGphor 3 system (GE Healthcare). For the second dimension, the IPG strips were first reduced for 15 min in SDS-equilibrium buffer (6 M urea, 50 mM Tris-HCl, pH 8.8, 30% glycerol, 2% SDS, trace of bromophenol blue and 0.5% (w/v) DTT) and then carbamidomethylated for 15 min in the same buffer containing 4.5% (w/v) iodoacetamide instead of DTT. The second dimension was performed on 12.5% polyacrylamide gels (20 × 24 cm) using the Ettan Dalt six system (GE Healthcare) at 10 mA per gel for 1 h, and then with an increased current of 15 mA per gel until the bromophenol blue band reached the end of the gel cassette. Electrophoresis was carried out under continuous cooling at 20°C.

### Image analysis and spot picking

The in-gel fluorescence was scanned on a Typhoon Trio (GE Healthcare) at appropriate wavelengths to detect Cy3 and Cy5 specific emission corresponding to the protein concentration in every single spot [38]. The intragel spot detection of the multiplexed gel images was performed using differential in-gel analysis (DIA) included in the DeCyder 6.5 software (GE Healthcare). The images for each gel were merged; spot boundaries were detected followed by a normalization of the spot volumes revealing differential spots. The gels were removed from the glass plates and stained with Flamingo Pink (Bio Rad) according to the manufactures instructions. The post-stained gels were mounted on a non-backed gel frame, scanned and analyzed using Image Master 6.0 (GE Healthcare). Selected spots were picked by an Ettan spotpicker (GE Healthcare) equipped with a 2 mm picking head according to the manufacturer's instructions. The picked gels were again scanned to verify the correct location of the cut spots.

### In-gel tryptic digestion and mass spectrometry

Gel pieces were washed twice with HPLC grade water (Roth, Karlsruhe, Germany) and dehydrated in a first step with 25 mM ammonium bicarbonate (ABC) in 50% acetonitrile (ACN) and shrunk in pure ACN as a second step, according to [39]. The dry gel pieces were then rehydrated with 100 ng sequencing-grade trypsin (Serva, Heidelberg, Germany) in 10 μl of 5 mM ABC. After rehydration, 10 μl of 5 mM ABC were added. For tryptic in-gel digestion, samples were incubated overnight at 37°C. To extract the peptides after the incubation period, 20 μl of 0.3% trifluoroacetic acid (TFA) in ACN were added and the samples were sonicated in a water bath (Sonorex Super RK100; Bandelin, Berlin, Germany) for 15 min. The liquid phases were collected, lyophilized, redissolved in 0.5 to 1 μl MALDI matrix solution (3,2 mg/ml α-cyanohydroxycinnamic acid (Sigma) dissolved in 65% ACN/0,1% TFA) and spotted onto a stainless steel 192-well MALDI plate and air-dried. The samples were analyzed by peptide mass finger printing in reflectron mode using the 4700 Proteomics Analyzer mass spectrometer (Applied Biosystems, Framingham, MA, USA). For MS analyses, typically 750 shots were accumulated for each spot. The peptide mass spectra were processed by internal calibration with autolytic fragments of porcine trypsin using the GPS Explorer software version 3.6 (Applied Biosystems) and searched against human proteins in the NCBI database (191694 entries, 2007/02/28, Homo sapiens) using MASCOT V2.0 (Matrix Sciences, London, UK).

### Database analysis

Database searches with MASCOT were performed using the following parameters: the modification on cysteine residues by amidomethylation was set as obligate, methionine oxidation was considered as a potential modification; the maximum number of missed tryptic cleavages was one; the monoisotopic masses were considered and the mass tolerance was set to ± 50 ppm.

## Authors' contributions

HS established the purification protocol, performed the DIGE experiments and prepared the manuscript. MN participated in sample preparation, DIGE experiments and Western blot analyses. CG and ML were responsible for the mass spectrometric analyses and respective parts of the manuscript. RL contributed the electron micrographs of individual lysosomal fractions. OJ designed and coordinated the study and wrote the manuscript. All authors have read and approved the final manuscript.
